# Mercury, Autoimmunity, and Environmental Factors on Cheyenne River Sioux Tribal Lands

**DOI:** 10.1155/2014/325461

**Published:** 2014-04-24

**Authors:** Jennifer Ong, Esther Erdei, Robert L. Rubin, Curtis Miller, Carlyle Ducheneaux, Marcia O'Leary, Bernadette Pacheco, Michael Mahler, Patricia Nez Henderson, K. Michael Pollard, Johnnye L. Lewis

**Affiliations:** ^1^College of Pharmacy, Community Environmental Health Program, University of New Mexico Health Sciences Center, 905 Vassar NE, Albuquerque, NM 87106, USA; ^2^School of Medicine, University of New Mexico Health Sciences Center, 1 University of New Mexico, Albuquerque, NM 87131, USA; ^3^Department of Natural Resources, Cheyenne River Sioux Tribe, P.O. Box 590, East Highway 212, Eagle Butte, SD 57625, USA; ^4^Missouri Breaks Industries Research, Inc., Hc 64 Box 52, Timber Lake, SD 57656, USA; ^5^INOVA Diagnostics, Inc., 9900 Old Grove Road, San Diego, CA 92131, USA; ^6^Black Hills Center for American Indian Health, 701 St. Joseph Street, Suite 204, Rapid City, SD 57701, USA; ^7^Scripps Research Institute, 10550 North Torrey Pines Road, La Jolla, CA 92037, USA

## Abstract

Mercury (Hg), shown to induce autoimmune disease in rodents, is a ubiquitous toxicant throughout Cheyenne River Sioux Tribe (CRST) lands. CRST members may be exposed to Hg through fish consumption (FC), an important component of native culture that may supplement household subsistence. Our goals were to ascertain whether total blood Hg levels (THg) reflect Hg exposure through FC and smoking, and determine whether THg is associated with the presence of anti-nuclear antibody (ANA) and specific autoantibodies (sAuAb). We recruited 75 participants who regularly consume fish from CRST waters. Hg exposure through FC and smoking were assessed via questionnaires. Whole blood samples were collected from participants, and THg was measured using ICP-MS. ANA and sAuAb in serum were modeled using demographic and exposure information as predictors. Female gender, age, and FC were significant predictors of THg and sAuAb; self-reported smoking was not. 31% of participants tested positive for ANA ≥ 2+. Although ANA was not significantly associated with Hg, the interactions of gender with Hg and proximity to arsenic deposits were statistically significant (*P* < 0.05). FC resulted in a detectable body burden of Hg, but THg alone did not correlate with the presence of ANA or sAuAb in this population.

## 1. Introduction

For more than a century, mining from greater than 900 mines in the Black Hills, including gold mines in which Hg was used for amalgamation purposes, has released contaminants into watersheds draining onto CRST lands [1]. Additionally, approximately one ton of airborne Hg is emitted per year from coal power plants in Montana, Wyoming, North Dakota, and South Dakota [2] and carried downwind to CRST lands where precipitation and dust wash this mercury out of the air into water and soil. Thus, Hg is virtually ubiquitous throughout the CRST reservation. Studies over the last decade conducted by the tribe, United States Environmental Protection Agency (USEPA), and University of Colorado [3] have documented high mercury concentrations in mid-flow water samples and sediment [4], invertebrates [5], and fish [5–7]. As a result of the widespread presence of Hg in the environment, fish consumption warnings have been posted along the Cheyenne River since 1974, yet no comprehensive health studies have ever been conducted in the CRST population to assess the health effects of consuming fish from tribal waters. In spite of posted warnings, CRST members still consume locally caught fish for complex reasons. Fishing and fish consumption are not only important in Lakota culture, but high rates of poverty (~50%) [8, 9] and unemployment (88%) [10] on the CRST reservation increase the community's likelihood of using fish to supplement household subsistence. Therefore, the safety of eating mercury-contaminated fish caught on tribal lands was a prime concern for CRST members. To address the CRST's environmental health concerns, a research partnership*, Environmental Justice on Cheyenne River*, was established in 2003 among the CRST Department of Environment and Natural Resources (DENR), the Black Hills Center for American Indian Health, and the University of New Mexico Community Environmental Health Program (UNM CEHP). Through community forums and discussions with tribal leaders, the partnership identified a major concern that a perceived increase in autoimmune disease (AD) prevalence in the CRST population might be related to Hg exposures through fish consumption, as well as a widespread frustration that actual health studies had not occurred in spite of Hg warnings posted for nearly 40 years. Although deidentified numbers of autoimmune cases were obtained from Indian Health Service (IHS) data sources, interpretation of the prevalence is difficult in identification of an appropriate denominator and determination of an appropriate comparison figure for Native American populations. Data on antinuclear antibody (ANA) prevalence in Native populations has not been evaluated. Prevalence of ANA in other US populations was recently derived from National Health and Nutrition Survey data (NHANES) [11, 12], but values for Native American populations could not be extracted due to no representation in that sample. Reference values for specific AD in tribal populations relative to the US total population are also not readily accessible.

Since tribal populations are comparatively more homogenous than other studied US populations, it may be tempting to ascribe any elevations in AD in the CRST merely to genetics. However, while genetic susceptibility has long been acknowledged as an important causative factor in the development of AD and evidence [13, 14] exists that genetic composition may predispose CRST members to AD, it is estimated that genetic factors only account for one-third of disease risk and that gene-environmental interactions play a vital role in the onset of autoimmunity [15]. The growing role of environmental factors, including aluminum metal compounds and thimerisol in vaccines, as adjuvants to the pathogenesis of autoimmunity has been studied extensively [16]. In addition, studies [17, 18] indicate that Hg toxicity and autoimmunity may be synergistically enhanced by various infectious and noninfectious triggers. It is reasonable that chronic stimulation of the immune system by environmental Hg may act through similar mechanisms. To address the community's concerns and begin to address existing gaps in knowledge about the effects of chronic low-level environmental exposures to metals, we sought to systematically examine the relationships among fish consumption, THg, and basic immune system markers in the CRST population in this study.

Existing knowledge about the effects of metals on the immune system comes mainly from the use of rodent models. In these models, relatively high doses of inorganic mercury administered to genetically susceptible mouse strains lead to the development of lupus-like autoimmune syndrome, which includes increased circulating antibodies to nuclear targets (antinuclear autoantibodies, ANA) [16, 17]. Further, exposure to inorganic* or* organic mercury exacerbates and accelerates the development of lupus-like disease in susceptible mouse strains [18–21]. Rodent models of mercury-induced autoimmunity [22–24], as well as their consistency with sex differences in autoimmune disease incidence observed in humans, suggest it is biologically plausible that Hg and other metals contribute to autoimmune pathogenesis in humans. Yet, with the exception of a few epidemiologic studies investigating the role of mercury amalgam fillings in multiple sclerosis [25, 26] and studies of ANA and cytokines in mercury-exposed Amazonian Brazil populations [27–30], too few [31, 32] have investigated the potential role of chronic environmental metal exposures as risk factors in the development of AD in humans. While relationships between metal exposure and immune dysfunction have been demonstrated in animals, limited data exist in humans. Since Hg has long been linked to development of AD-like symptoms in animal models [17], we hypothesized that increased mercury exposure, primarily through fish consumption, would be associated with higher levels of circulating autoantibodies in the CRST population. In order to test this hypothesis and respond to community concerns, we modeled ANA and specific autoantibody concentrations in blood collected from CRST community members using THg, fish consumption, smoking, age, gender, and proximity to high-concentration arsenic sediment deposits as predictors.

## 2. Materials and Methods

### 2.1. Human Subjects

The protocol and study design were approved by the Executive Committee of the Cheyenne River Sioux Tribe Tribal Council (Tribal Resolution number: E-302-08-CR and extended under E-343-2009-CR) and by the University of New Mexico Health Sciences Center Human Research Protection Office (HRPO number: 08-486). As deidentified serum samples were sent to the Scripps Research Institute Department of Molecular and Experimental Medicine, the Scripps Research Institute's Institutional Review Board provided approval for an analysis of serum ANA and specific autoantibodies.

Participants were recruited by using community-based communication tools and procedures previously developed by this team and applied in the* Environmental Justice on Cheyenne River* study. Outreach, enrollment, and sampling were conducted in conjunction with local collaborators, notably Missouri Breaks Industries Research, Inc. (MBIRI), who were crucial contributors in several previous federally funded research projects among Cheyenne River Tribal communities, and collaborating staff from the CRST DENR. The recruitment was targeted toward fishermen and their family members, who were known to local collaborators as regular consumers of fish caught from the Cheyenne River and its tributaries.

**Figure 1:**
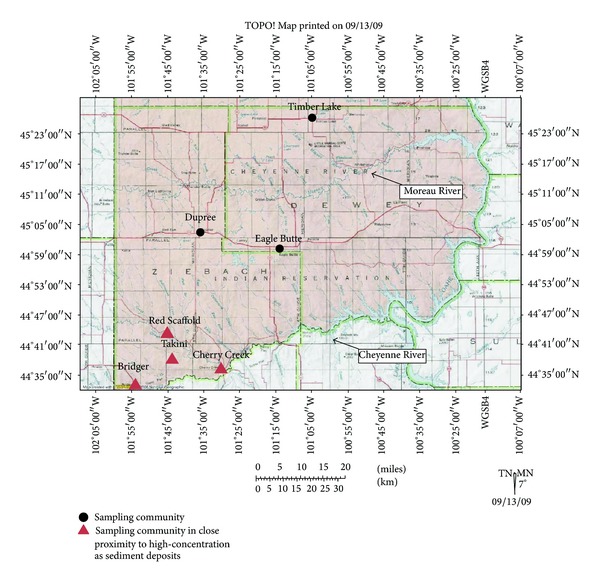
Map of Cheyenne River Sioux Tribal lands and sampling communities.

Written informed consent was obtained from a total of 75 adults living on the CRST Lands during the peak of fishing season. The study population includes members from multiple communities including Eagle Butte, Cherry Creek, Dupree, Timber Lake, Red Scaffold, Bridger, Takini, and Howes (Figure 1). At each location, enrollment was conducted and biological samples were collected in community centers. These communities, some of which are in close proximity to rivers, lakes and ponds on CRST lands, encompass both commercial centers and rural areas, as well as members whose primary source of food is store-bought versus acquired from the local environment (subsistence lifestyle), and therefore reflect a wide range of potential exposures to Hg through fish consumption. Smoking status was a concern as an alternate contributor to THg based on previosly reported increases in smoking on the CRST reservation [33] and the demonstrated contributions to THg from cigarette smoking [34, 35]. MBIRI team interviewers collected demographic (e.g., age, gender), health condition, fishing, and smoking habit information through personal interviews conducted in English using a Centers for Disease Control- (CDC-) developed fish consumption survey and our own short smoking exposure questionnaire. When participants needed information or clarification spoken in their native language, the community-certified nurse interviewers provided the answers.

### 2.2. Surveys Used in the Study

#### 2.2.1. Fish Consumption

A CDC questionnaire, as well as local collaborators' knowledge of CRST community members' fishing habits, was used to assign a categorical rating of 1, 2, or 3 to each participant's fish consumption, with 1 designating minimal to no fish consumption, and 3 corresponding to high fish consumption. For reference to the local environment and consumption patterns, the safe amount of fish intake per month was previously recommended by our* Environmental Justice on Cheyenne River* study using DENR Hg measurements from local fish and USEPA guidelines [37]. One monthly recommended serving was defined as one northern pike, two bass or perch, three walleye, or four catfish. A rating of (1) denotes consumption of <1 serving of fish per month; (2) denotes 1-2 servings/month; and (3) denotes >2 servings/month.

#### 2.2.2. Smoking

To account for smoking as both a potential source of Hg and contributor to immune system effects, participant smoking data were collected via questionnaire. The questionnaire was based on coauthor PNH's previous work [33] on smoking among tribal members and was given to all participants in order to obtain self-reported information regarding smoking exposures. The questionnaire encompassed both direct and second-hand exposure to cigarette smoke. There were seven questions total; smoking score was coded as low (1) when fewer than two questions were answered affirmatively; medium (2) when 3-4 questions were answered affirmatively; and high (3) when greater than five questions were answered affirmatively. A participant was considered an “active smoker” if he/she answered “yes” to the included question, “Do you smoke currently?”

#### 2.2.3. Arsenic Proximity

**Figure 2:**
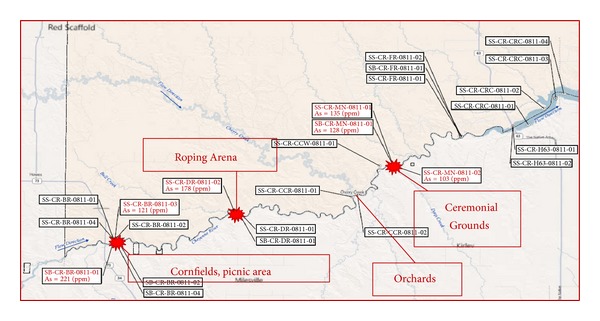
Map of arsenic sampling conducted by USEPA and CRST DENR. Concentrations of arsenic in sediment exceeding 100 ppm are marked with a burst pattern. Exposure-relevant sites are labeled with activities frequently conducted in those areas.

During the analytic phase of this study, elevated sedimentary arsenic deposits were discovered in land-use areas in close proximity to several of the sampling-site communities in this study (Figure 2). Ongoing collaborations among DENR, Dr. Lewis, and USEPA Region 8 are surveying residents and characterizing exposure pathways, frequencies, and duration. However, as these deposits were identified subsequent to consent for this study, no arsenic biomonitoring data were obtained from the population in the original design nor were exposure activities involving these sedimentary deposits identified. Due to studies in humans and animals indicating that arsenic suppresses autoimmunity [38, 39], while mercury may either suppress or increase autoimmune response [28, 32], a surrogate of participant arsenic exposure was incorporated into models to address potentially competing exposures. A binary surrogate for arsenic exposure was derived; the designations of “near” or “far” proximity to known quantified environmental arsenic deposits by USEPA were given according to self-reported participant residence data. The designation of “near” was given to participants who live in the communities of Cherry Creek, Takini, Bridger, and Red Scaffold (Figure 1). Surveys of residents have identified potential exposure pathways which include common land-use practices such as fishing; herb, fruit, and firewood gathering; inhalation of wood combustion products during sweat lodge and ceremonial practices; and roping/other horseback riding activities along the Cheyenne River near the identified alluvial arsenic deposits (Figure 2) (personal communication C. Ducheneaux and J. Lewis). Participants residing in the Eagle Butte, Dupree, and Timber Lake (Figure 1) communities more distal to the arsenic deposits were given a designation of “far” for arsenic proximity in this pilot assessment. This binary variable was incorporated to determine if further studies on the relationship of these exposures to AD were warranted.

### 2.3. Biological Sample Collection

#### 2.3.1. Blood and Serum Samples

Venous blood samples were collected by venipuncture at community centers or during home visits by a trained and certified phlebotomist or registered nurse. One red top (9 mL) for serum collection and one purple top (7 mL) Vacutainer tube were collected for biomonitoring from each participant. After clotting, serum samples were spun at 2,500 rpm for 10 minutes and separated into cryovials and placed into a −80°C freezer. At a later time point, sera were shipped to the UNM HSC laboratory and subsequently to the Scripps Research Institute.

### 2.4. Experimental Use of Collected Biological Samples

#### 2.4.1. Biomonitoring

The EDTA-containing whole blood samples were transported to the CDC ONDIEH/NCEH Environmental Health Laboratory where inductively coupled plasma mass spectrometry (ICP-MS) was used to determine THg concentrations. The limit of detection was 0.32 *μ*g/L.

#### 2.4.2. Detection of Autoantibodies

**Figure 3:**
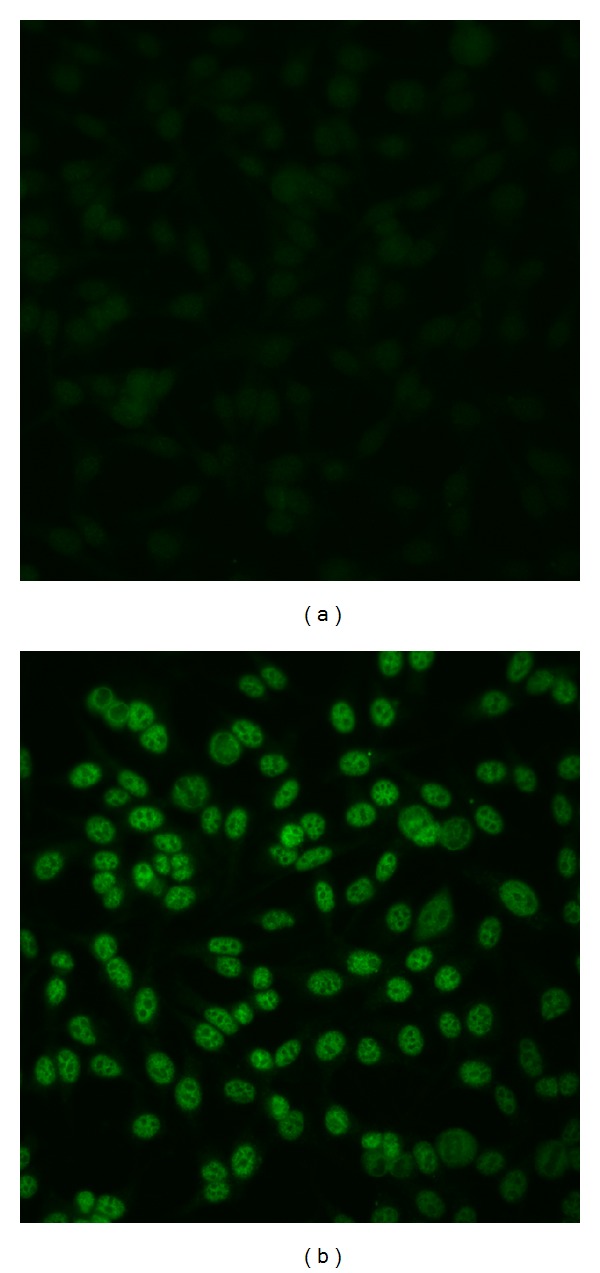
Examples of ANA determination by immunofluorescence. Human sera were incubated with HEp-2 cells followed by fluorescent anti-human IgG. The sample on the left (a) is ANA negative, while the sample on the right (b) was considered 2+ ANA positive, showing fine speckled nuclear staining sparing the nucleolus.

*(1) Antinuclear Antibodies (ANA)*. The presence of ANA was determined by indirect immunofluorescence (IIF) microscopy using HEp-2 cells as substrate (MBL-BION, Des Plaines, IL) and Alexa Fluor 488 Goat Anti-Human IgG (H + L) (Life Technologies, NY, USA) as detecting reagent. Sera were diluted 1 : 100 in serum diluent, and detecting reagent 1 : 200 with anti-Ig diluent as previously described [40]. Slides were viewed by a single observer (KMP) blinded to participant identity on a BH2-RFCA fluorescence microscope (Olympus, Lake Success, NY). Intensity of fluorescence was graded on a scale of 0–4+. A reading of ≥2+ was considered significant and further used in our statistical modeling. This cut-off value reflects a stricter value based on the literature [36, 41]. Example immunofluorescence images for ANA determination can be found in Figure 3 for negative (0) and ANA ≥ 2+ readings.

*(2) Specific Autoantibodies (sAuAb)*. Commercially available kits (INOVA Diagnostics, San Diego, CA) were used as described by the manufacturer to detect and quantify serum autoantibodies to the following antigens: chromatin, Sm, RNP, SSA, SSA-52, SSB, Scl-70, RNA Pol III, CENP-A/B, Ribo-P, Jo-1, M2 EP (MIT3), and primary biliary cirrhosis (PBC) screen, a panel of antigens (M2 EP, gp210 and sp100 IgG/IgA). Assay-specific positive controls were used to convert optical density values to units in order to determine whether the results of assays for Sm, RNP, SSA, SSA-52, SSB, Scl-70, RNA Pol III, Ribo-P, and Jo-1 were negative/equivocal (<20 units), weakly positive (20–39 units), moderately positive (40–80 units), or strongly positive (>80 units). The tests for M2 EP and the PBC screen were interpreted as being equivocal from 20.1 to 24.9 units and positive for >25 units. Centromere-A/B (CENP-A/B) has negative/equivocal results for <20 units, weak positive for 20–30 units, and strong positive for >30 units. Chromatin has a negative/equivocal reading <20 units, moderate positive between 20 and 60 units, and a strong positive >60 units.

Additional assays to chromatin, denatured DNA (single-stranded, dDNA), native DNA (nDNA), and histones were quantified by enzyme-linked immunosorbent assays (ELISA) as previously described in [42, 43]. Briefly, Immulon 2HB microtiter plates (Dynex Laboratories, Inc., Alexandria, VA) were coated with antigen at 2.5 *μ*g/mL concentrations. For the antichromatin assays, in-house-prepared H1-stripped chromatin was used as the solid-phase antigen. S1-nuclease (Invitrogen-) treated DNA (Calbiochem) was used in the antinative DNA assay, and DNA was heated for 10 min and then quickly cooled for preparation of the dDNA antigen. Prior to coating plates with nDNA or dDNA, plates were pre-coated at 2 *μ*g/mL with the synthetic 20–50 kDA polypeptide poly(lys-phe) (Sigma-Aldrich), comprising a co-polymer of lysine and phenylalanine at a 1:1 ratio as previously described [43]. Total histone was from Worthington. Serum samples were diluted 1 : 200 and incubated on the plate for 2 hours at room temperature with gentle shaking. Each sample was run in duplicate. The bound antibodies were detected with peroxidase-conjugated anti-human IgG (Southern Biotech, AL) and 2,2′-azino-bis(3-ethylbenzthiazoline-6-sulfonic acid) (MP Bioproducts) as the secondary substrate. Optical densities (OD) beyond the range of direct measurement at 1 h in the ELISA were extrapolated from OD at earlier time-points as described [44]. Positive and negative control sera were always included in each assay, and values determined in different assays were normalized by multiplying by the ratio of the reactivity of the positive control sera tested in each assay.

### 2.5. Statistical Analysis: Modeling

Total blood mercury results lower than the limit of detection (LOD = 0.32 *μ*g/L) were analyzed with the value of $\text{LOD}/\sqrt{2}$, because fewer than 50% of participants had a biomonitoring value < LOD. Total blood mercury results are presented as median values with the interquartile range, since the median is a better indicator of the true population value for the distribution of the collected data. The mean and 95% confidence interval for THg are also presented for ease of comparison with published NHANES population data. When comparing groups (e.g., male versus female) in relation to THg and ANA status, Fisher's exact test was used.

To characterize the complex exposures on CRST lands and their relationships to immune system responses and autoantibody production, several statistical models incorporating biomonitoring data, fish consumption score, smoking exposure score, distance to arsenic contamination, and immune system markers were developed. The approaches included multiple linear, logistic, and Poisson regression models to evaluate relative contributions of environmental exposures to circulating autoantibodies. They also included accepted risk factors such as age and gender. Multiple linear regression was used to model THg in relation to environmental exposure and risk factors, while logistic regression was used to model ANA ≥ 2+ in relation to predictors. Poisson regression was used to model the numbers of specific autoantibodies (both determined via INOVA and additional assays) with environmental exposure and risk factors. Poisson models accommodate count information with nonnormal distribution, thereby enhancing the analytical capacity to understand the exposure factors' underlying contribution to risk. Full models were fitted using all demographic, biomonitoring, and exposure data as predictors. Reduced models were selected using the Akaike information criterion (AIC), which is a measure of the relative quality of a statistical model. The openly available statistical software R [45] and the stepAIC function from the package MASS [46] were used to complete this AIC model selection, where

(1) $$\begin{array}{c} \text{AIC}=2k-2\ln\left(L\right). \end{array}$$

And *k* = 2 and *L* is the likelihood of each model. The AIC selection criterion minimizes the distance between the predicted values of the model and the true values while also favoring models with fewer parameters.

#### 2.5.1. Specific Approach to Analyze Specific Autoantibody Results from INOVA Assays

Since positivity for individual-specific autoantibodies was expected to be lower in frequency, we pooled all participants who tested positive for any specific autoantibodies to examine an overall prevalence of specific autoantibodies and the contributing exposure factors by summing the number of specific autoantibodies for which each participant tested positive and then conducting Poisson regression on the count variable generated. Biomonitoring data and exposure data were incorporated as well. This count variable makes biological sense because it follows established clinical AD diagnosis criteria; individuals diagnosed with AD present with varying combinations of specific autoantibodies.

#### 2.5.2. Specific Approach to Analyze Specific Autoantibodies Results from Additional Autoantibody Assays

Poisson regression was used to model several combinations of autoantibodies that would otherwise be rarely detected. The combinations modeled were selected in order to examine different possible scenarios of positive autoantibody response. Individuals with detectable autoantibody response were classified into groups according to the following scenarios based on the literature [42–44]:

- presence of any autoantibody response (nDNA, dDNA, histone, chromatin);
- detectable levels of potentially environmentally related autoantibodies (dDNA and histone);
- disease-associated autoantibodies (nDNA and chromatin).

Results of these models were summarized in several tables presented in the next section.

Because anti-chromatin autoantibodies were detected using both INOVA and in-house assays, we evaluated the reproducibility of this antigen. We applied a nonparametric correlation (Spearman *r*-value) and used a *z*-score.

#### 2.5.3. Reporting of Significant Results

While our primary results will follow a more standard reporting cutoff of *P* < 0.05, we will report those with probabilities up to 0.1 to guard against Type 2 error and to ensure comprehensive consideration of predictors in designing follow-up investigations. This decision is warranted given (1) the importance of the results to the communities, (2) the lack of prior studies in this area and in this population, and (3) the lack of biomonitoring data at this time on other potential environmental exposures including arsenic resulting in imprecise measures for that variable.

## 3. Results

### 3.1. Mercury Exposure and Population Characteristics

**Figure 4:**
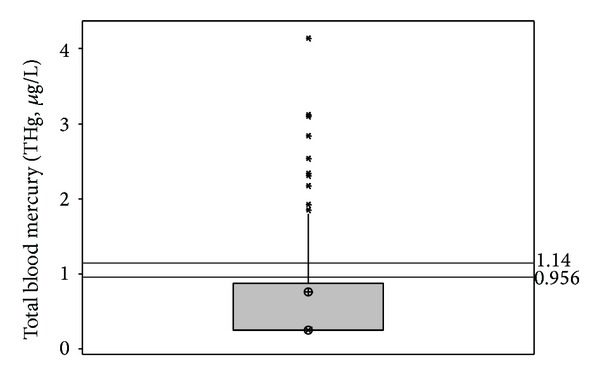
Scatterplot of total blood mercury (THg) for sample population with median denoted by an encircled “X” and mean denoted by an encircled cross. The reference lines at 0.956 *μ*g/L and 1.14 *μ*g/L indicate the 95% CI for THg in the US population from NHANES [36].

Population characteristics, including gender, smoking score, fish consumption score, community size, and proximity to identified high-concentration sedimentary arsenic deposits, are summarized in Table 1. Total blood mercury concentrations (THg) ranged from below the limit of detection (LOD, 0.32 *μ*g Hg/L) to 4.14 *μ*g Hg/L, with a median lower than the LOD (Figure 4). For most population characteristic categories, the median THg was below the LOD, with the exception of males (0.37 *μ*g Hg/L) and participants with “medium” or “high” fish consumption scores (0.35 and 0.54 *μ*g Hg/L, resp.).

Total blood mercury in the CRST depended on gender, age, and fish consumption but not smoking. The reduced multiple linear regression modeling results for THg as a response with demographic and exposure information as predictors are summarized in Table 2. Male gender and older age were significant predictors of THg (*P* = 0.0084 and 0.022, resp.); fish consumption approached significance as a predictor for THg (*P* = 0.053).

### 3.2. Prevalence of ANA in the CRST Population

Anti-nuclear antibodies (ANA) were analyzed in serum samples from all participants. Data are presented in Table 1 and representative images of negative and ANA ≥ 2+ readings are shown in Figure 3. Approximately thirty-one percent of participants had an ANA reading of ≥2+. For readings ≥2+, ANA prevalence was significantly higher in women than in men (24% versus 5%; *P* = 0.025). ANA prevalence was also larger in community members living in proximity to high-concentration sedimentary arsenic deposits (30% versus 8%; *P* = 0.028).

Gender and fish consumption were significant predictors of ANA ≥2+, and gender modifies the effects of environmental exposures with respect to ANA. The logistic regression model information is shown in Table 3. Age and fish consumption are borderline predictors (*P* < 0.10) of ANA ≥ 2+ (*P* = 0.081 and *P* = 0.092, resp.), with age and fish consumption positively associated with the probability of ANA ≥ 2+ level of circulating ANA. Gender, THg, and proximity to arsenic, by themselves, do not strongly correlate with the probability of ANA ≥ 2+; however, the* interactions* of gender with THg and arsenic proximity are significant, and their odds ratios are greater than one (OR = 13.83, *P* = 0.026 and OR = 27.71, *P* = 0.04, resp.).

### 3.3. Specific Autoantibodies in the CRST Population

**Figure 5:**
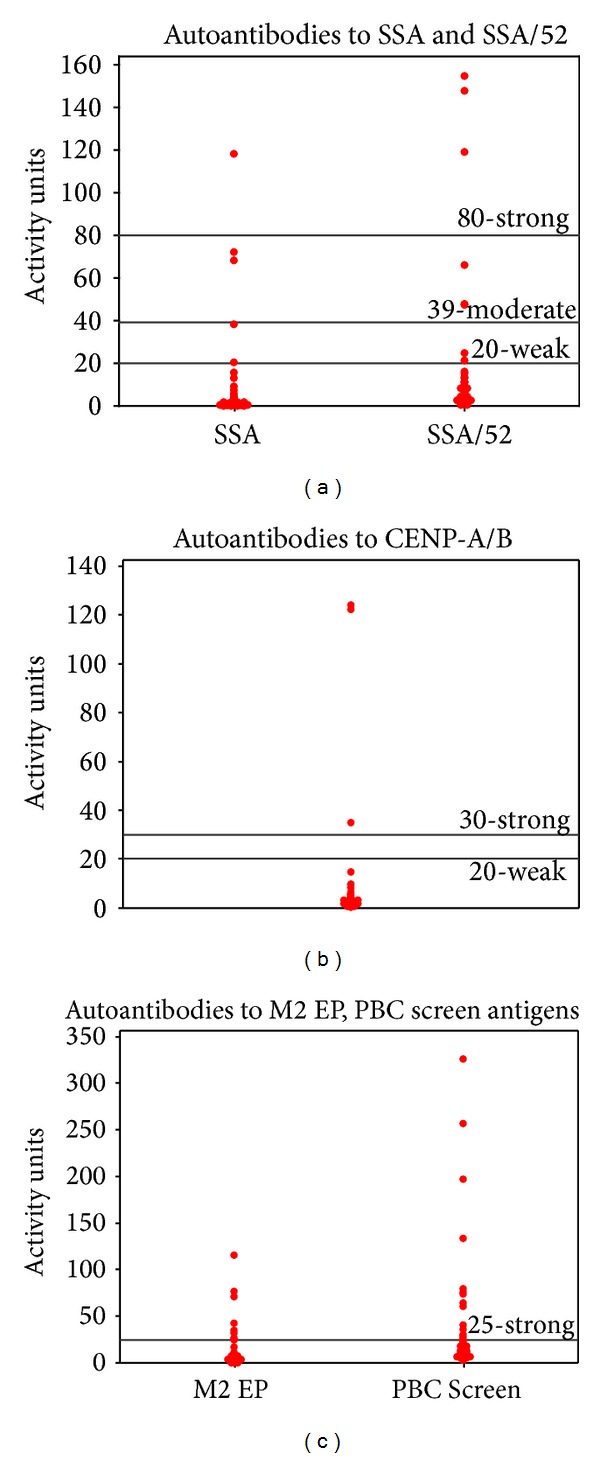
Dot plots of detectable autoantibodies in participant serum measured in activity units with labels for clinical cutoffs. Note: In clinical practice, there are no “moderate” positive readings for CENP-A/B; there are no “weak” or “moderate” positive readings for M2 EP and the PBC Screen.

Of the specific autoantibodies for which participant sera were tested, SSA, SSA/52, CENP-A/B, M2 EP, and the autoantibodies detected by the primary biliary cirrhosis (PBC) panel were noteworthy. These results are summarized in Figure 5 and Table 4. Fifteen percent of participants tested positive for autoantibodies to M2 EP, while 24% were positive for autoantibodies to the PBC panel.

The number of specific autoantibodies detectable by INOVA kit increased with female gender and fish consumption score. Information for the reduced Poisson model for the number of detectable specific autoantibodies using INOVA assays can be found in Table 5(a). The number of specific autoantibodies detectable from INOVA assays was associated significantly with female gender (*P* = 0.0064). The model indicated that the mean number of specific autoantibodies detectable in serum is increased by a factor of 6.5 in female versus male community members. Age and fish consumption had significant (*P* = 0.012 and *P* = 0.0073, resp.) but smaller effects on the number of specific autoantibodies in the collected serum samples. In particular, the mean number of specific autoantibodies detectable by INOVA assay was 2.6 times greater in participants with a high (3) versus a low (1) fish consumption score. The number of participants positive for autoantibodies to native DNA, histone, and chromatin using in-house assays was small in our study. No significant associations were found between any demographic or exposure predictors, including smoking, and various combinations of in-house autoantibodies except in the case of dDNA and histone. The values for the reduced model can be seen in Table 5(b). Fish score was a significant predictor (*P* = 0.035) of the number of subjects with elevated anti-dDNA and anti-histone autoantibodies detectable using in-house assays. An increase of one fish score category predicts a 2.5-factor increase in the number of dDNA and histone autoantibodies. Smoking was a borderline predictor (*P* = 0.065) with a 0.4-factor decrease in the number of dDNA and histone autoantibodies for an increase of one smoking category.

Antichromatin positivity and reproducibility between INOVA and additional assays were confirmed in all positive serum samples (5/75); there was 100% agreement in detection using INOVA and in-house assays.

## 4. Discussion

We assumed that, due to consumption of locally caught fish, community members would have elevated levels of total blood mercury (THg). We hypothesized that THg would correspond to an increased level of autoantibodies, as has been shown in animal models [17]. Contrary to expectations, although Hg deposition in fish tissue had been documented in CRST sources by DENR, the detected THg levels in participants were low with a median THg < LOD, despite sampling during the middle of fishing season when fish consumption was considered to be maximal. The median THg for all participants (<LOD) was lower than the published results of the NHANES survey [47]. NHANES reported a mean THg of 0.944 *μ*g/L for those 12 years and older [47]. Native American populations were not stratified in that study; the data were compiled under “other” ethnicity. The low levels of blood mercury among the CRST members found in our study confirmed the THg levels reported in a 2008 collaborative study between our team and CDC [48, 49]. Potential reasons for the observed low THg include variation due to race/ethnicity and possible physiological and metabolic changes among CRST community members, or possible alterations in deposition and clearance with repeated exposure in this population.

While gender, age, and fish consumption showed an impact on THg levels in the CRST population, smoking did not (Table 2). This finding is puzzling since 56% of the participants reported current smoking, yet only 45% of that group had THg above the detection level. The trends in our data parallel those seen in the NHANES survey [50]. Males have greater mean THg versus females, and THg increases with age. Males may consume larger quantities of locally caught fish or engage in activities that increase dust and particulate exposures to mercury (e.g., agricultural work, horse-tending). The age-dependent increase in THg found in this study, as well as in Wolkin et al. [48] and the NHANES survey [50], is likely due to the accumulation of metals in the body over time.

Thirty-one percent of participants had an ANA reading of ≥2+. ANA production could be associated with chronic toxicant exposure, which introduces self-antigens to antigen presenting cells, resulting in the breakdown of T-cell tolerance. While no single predictor was significantly associated (*P* < 0.05) with ANA ≥ 2+, fish score was a borderline predictor (*P* = 0.092). A larger proportion of ANA-positive participants were female, which concurs with the literature and clinical findings about autoimmune diseases [24], and may support a possible role for female hormones in AD and immune dysregulation. Although THg and proximity to high-concentration arsenic deposits, by themselves, did not correlate with the probability of ANA ≥ 2+, the* interactions* of female gender with THg and female gender with arsenic proximity are significant (*P* = 0.026 and *P* = 0.040, resp.) and the odds ratios were large (OR = 13.8 and 27.1, resp.). Gender differences may reflect alterations in the molecular mechanisms by which gender-specific detoxification occurs within the human body.

Another interesting finding is that current smokers were less likely to have ANA ≥ 2+ results (Table 1). Additionally, the specific autoantibody model estimates for smoking were negative with ORs less than one (Tables 5(a) and 5(b)), suggesting a protective effect of smoking. The fact that fewer autoantibodies were detected in this subgroup of smokers sheds light on probable molecular mechanisms by which smoking induces immunosuppressive effects.

There were significant associations between predictor exposure variables and the presence of autoantibodies to dDNA and histone (Table 5(b)). This is potentially similar to previously observed instances of xenobiotic-induced antibody responses such as drug-induced lupus [42]. Autoantibody production to dDNA and histone may also be linked to epigenetic changes triggered by environmental stimuli.

The CRST population exhibited strong positivity for M2 EP autoantibodies and autoantibodies detectable with the PBC screen, both of which are associated with liver diseases. It is possible that the medical problem of high rates of idiopathic liver cirrhosis in Sioux communities (personal communication J. Henderson) may have environmental etiology. Similar findings were reported among Alaskan Natives [51]. As with ANA ≥ 2+ models, fish score was a significant predictor of* specific* autoantibodies using both detection methods (*P* < 0.01 for INOVA kit detection and *P* < 0.05 for in-house ELISA).

As to the specific mechanisms responsible for mercury and metal/metalloid-induced autoimmune responses in the CRST population, several mechanisms should be considered. In susceptible individuals, environmental metals may behave as adjuvants that prolong or enhance antigen-specific immune response through various mechanisms such as molecular mimicry [52], polyclonal activation of B cells [53], bystander activation [54], and epitope spreading [55]. Additionally, chronic exposures to environmental metals, including Hg and arsenic, are well known to induce oxidative stress. As has been characterized with thimerisol [56], this oxidative stress could lead to sensitization of inositol 1,4,5-triphospate (IP3) receptors, resulting in enhanced intracellular calcium release and subsequently the dysregulation of immune cells and autoimmunity. Another possibility includes the role of chronic gut exposures to ingested dietary nanoparticles of soil and minerals, which induce inflammasome production and the breakdown of immune tolerance via enhanced gastrointestinal antigen presentation. Since fish consumption was an important predictor of antibody production in this study, dietary exposure may be one potential pathway through which molecular markers of autoimmunity are generated, especially among native community members who are more likely to inhale and ingest large quantities of dust and metals due to their rural location, cultural practices, and subsistence and agricultural activities.

We hypothesize that fish consumption reflects multiple exposures, including coexposures to mercury, arsenic, and other environmental toxicants, such as pesticides, pharmaceuticals, and infectious agents. In animal and cell studies, Hg toxicity and autoimmunity are synergistically enhanced by coexposure to additional xenobiotics. These ideas will be explored in future studies, and additional activities that increase inadvertent exposure to toxicants will also be examined. Future studies will include a larger sample size, participant AD medical record history, and biomonitoring for arsenic and cotinine (indicator of smoking exposures) in order to address this study's limitations. We also acknowledge that technical issues with indirect immunofluorescence assays (IIFA) for the detection of ANA limit the comparability of these data to other population information and previous publications. However, IIFA is the gold-standard technique for ANA detection [57], and we attempted to minimize variability by using only one evaluator of staining (KMP, coauthor).

In this study, compelling evidence that the CRST population exhibited elevated levels of both ANA and specific autoantibodies was found. The observed results highlighted environmental toxicants that may contribute to autoantibody production in this population and also underscored the need to characterize the CRST communities' lifestyles and behaviors to better understand how complex exposures contribute to autoimmune health effects. There is a large knowledge gap concerning environmental influences on the development of AD, and it is imperative that they be addressed within the context of environmental health disparities issues, particularly in tribal communities. Information will empower CRST community members and leaders by aiding them in making informed decisions about health, health services, the environment, and the preservation of their culture, in which fishing plays a vital role.

## Figures and Tables

**Table 1:** Biomonitoring and ANA ≥2+ results linked with study participant characteristics.

Population characteristic	*N*	Participants with THg >LOD*	Hg biomarker median (interquartile range)	Hg biomarker mean (95% CI)	ANA reading ≥2+ (*n*, %)
All participants	75	36 (36%)	<LOD (<LOD–0.87)	0.75 (0.55–0.95)	23 (31%)

Gender					
Male	38	23 (61%)	0.37 (<LOD–1.81)	1.01 (0.67–1.37)	2 (5%)
Female	37	13 (35%)	<LOD (<LOD–0.56)	0.48 (0.32–0.63)	9 (24%)
Smoking score					
(1) Low	32	17 (53%)	0.37 (<LOD–0.74)	0.67 (0.41–0.92)	8 (25%)
(2) Medium	18	8 (44%)	<LOD (<LOD–1.24)	0.82 (0.36–1.28)	3 (17%)
(3) High	25	11 (44%)	<LOD (<LOD–1.21)	0.81 (0.39–1.23)	7 (28%)
Active smoker					
Yes	42	19 (45%)	<LOD (<LOD–0.96)	0.77 (0.49–1.05)	4 (10%)
No	31	16 (52%)	0.37 (<LOD–0.87)	0.76 (0.40–1.06)	7 (23%)
Fish score					
(1) Low	41	17 (59%)	<LOD (<LOD–0.60)	0.59 (0.36–0.82)	7 (17%)
(2) Medium	18	10 (56%)	0.35 (<LOD–1.53)	0.90 (0.38–1.43)	2 (11%)
(3) High	16	9 (56%)	0.54 (<LOD–1.89)	0.99 (0.51–1.48)	2 (13%)
Arsenic proximity					
Yes	23	7 (30%)	<LOD (<LOD–0.52)	0.45 (0.25–0.64)	7 (30%)
No	52	29 (56%)	0.37 (<LOD–1.27)	0.89 (0.62–1.16)	4 (8%)

*LOD = 0.32 *μ*g/L.

**Table 2:** Reduced model (multiple linear regression) for total blood mercury.

	Estimate	*P* value	Std. error
Intercept	0.412	0.46	0.56
Gender*	−0.545	*0.0084 *	0.2
Age	0.0181	*0.022 *	0.0077
Smoking score	−0.0127	0.91	0.12
Fish score	0.246	0.053	0.13
Arsenic proximity	−0.526	*0.020 *	0.22

*P* values less than or equal to 0.05 are italicized.

*Male gender was used as the reference, so the estimate describes the effect of being a female.

**Table 3:** Point estimates and 95% confidence intervals (CI) for coefficients and odds ratios (OR) for fitting logistic regression models for ANA ≥2+.

	OR	95% CI	*P* value
Intercept	0.011	N/A	0.93
Age	1.1	0.96–1.17	0.081
Gender	2.4	0.046–645.5	0.37
THG	0.4	0.045–1.75	0.89
Fish score	2.9	0.56–20.70	0.092
Gender: THG	13.8	0.97–487.8	*0.026 *
Gender: fish score	0.1	0.0013–1.12	0.97
Gender: arsenic proximity	27.1	0.68–2101	*0.040 *
Arsenic proximity	0.3	0.015–4.26	0.82

*P* values less than or equal to 0.05 are italicized.

**Table 4:** Results of selected specific autoantibody results from the CRST population sample.

*n* = 75	Negative	Moderate positive	Strong positive	Total positive
Autoantibody				
SSA	72 (96%)	2 (3%)	1 (1.3%)	3 (4%)
SSA-52	70 (93%)	2 (3%)	3 (4%)	5 (7%)
CENP-A/B	72 (96%)	0 (0%)	3 (4%)	3 (4%)
M2 EP	64 (85%)			11 (15%)
PBC panel	57 (76%)			18 (24%)

**Table:** (a)

	Factor of change	95% CI	*P* value
Intercept	0.0	0.00–0.18	0.0012
Gender*	6.5	1.69–24.78	*0.0064 *
Age	1.0	1.01–1.04	*0.012 *
Fish score	1.6	1.13–2.16	*0.0073 *
Smoking score	1.9	0.60–5.77	0.29
Gender: smoking score	0.5	0.25–1.01	0.053

*P* values less than or equal to 0.05 are italicized. *Male gender was used as the reference, thus, the factor of change reflects the effect of being female.

**Table:** (b)

	Factor of change	95% CI	*P* value
Intercept	0.2	0.004–9.831	0.406
Age	1	0.919–1.055	0.658
Smoking	0.4	0.127–1.064	0.065
Fish score	2.5	1.067–5.980	*0.035 *
Arsenic proximity	0	0.000–3.752	0.117
Age: arsenic proximity	1.1	0.984–1.244	0.092

*P* values less than or equal to 0.05 are italicized.
